# Exploration of the risk factors of essential hypertension with hyperhomocysteinemia: A hospital-based study and nomogram analysis

**DOI:** 10.6061/clinics/2021/e2233

**Published:** 2021-01-11

**Authors:** Jufang Wang, Jinman Du, Rui Fan

**Affiliations:** I Medical quality management office, Ningbo Medical Center Lihuili Hospital, Ningbo, Zhejiang 315040, China; II Physical examination center, Ningbo Medical Center Lihuili Hospital, Ningbo, Zhejiang, 315040, China

**Keywords:** H-type hypertension, risk factors, hospital-based study, nomogram

## Abstract

**OBJECTIVES:**

To explore the risk factors of essential hypertension with hyperhomocysteinemia (H-type hypertension) and design a nomogram to predict this risk.

**METHODS:**

A hospital-based study was conducted on 1,712 individuals, including 282 patients with H-type hypertension, 105 patients with simple hypertension, 645 individuals with hyperhomocysteinemia, and 680 healthy controls. Logistic regression and nomogram models were applied to evaluate the risk factors.

**RESULTS:**

Logistic regression showed that advanced age, male sex, high body mass index (BMI), high total cholesterol levels, high glucose levels, and high creatinine levels were risk factors of H-type hypertension in the healthy population and were integrated into the nomogram model. Advanced age, male sex, high BMI, high total cholesterol levels, and high glucose levels were shown to be risk factors of H-type hypertension in the hyperhomocysteinemia population. Male sex and high creatinine levels were shown to be risk factors of H-type hypertension in the hypertension population. Nomogram analysis showed that the total factor score ranged from 106 to 206, and the corresponding risk rate ranged from 0.05 to 0.95.

**CONCLUSIONS:**

Men are more likely to have H-type hypertension, and advanced age, high BMI, high total cholesterol levels, and high glucose levels are risk factors of H-type hypertension in healthy and hyperhomocysteinemia populations. Furthermore, high creatinine level is a risk factor of H-type hypertension in healthy and hypertension populations. Nomogram models may be used to intuitively evaluate H-type hypertension risk and provide a basis for personalized interventions.

## INTRODUCTION

H-type hypertension, a type of essential hypertension with hyperhomocysteinemia (HHcy), is a major risk factor of cardiovascular and cerebrovascular diseases, especially stroke (1-3). It has been reported that the risk of incident stroke is 12.7 times and that of stroke-related death is 11.7 times in patients with H-type hypertension compared with those in healthy controls (4). As a common complex chronic disease, the association between high homocysteine (Hcy) levels and high blood pressure may be concomitant and affected by numerous factors such as age, sex, body mass index (BMI), and biochemical indicators (5,6). Therefore, an accurate and intuitive assessment of the different effects of multiple risk factors on blood pressure and Hcy levels, is particularly important for implementing personalized interventions.

Previous studies have mainly used logistic models to explore the risk factors of H-type hypertension. Wang et al. demonstrated that age, sex, BMI, physical activity, smoking history, and low-density lipoprotein (LDL) cholesterol levels are associated with H-type hypertension (1). Furthermore, Han et al. indicated that age, smoking, sodium intake, systolic blood pressure, uric acid levels, triglyceride levels, and creatinine levels are associated with high Hcy levels in patients with hypertension (7). However, these studies were limited due to the complexity and technicality related to interpretation of the results, and as a result, the association between risk factors and H-type hypertension in healthy, HHcy, and hypertension populations remains under-researched.

Nomograms graphically express the numerical relationship between a disease and risk factors (8). Using nomogram models, healthcare workers can predict the incidence rate or survival rate of diseases according to a scoring system without having to use a complex formula for calculations (9). Nomograms have been used in the risk assessment of cancer, diabetes, dyslipidemia, and hypertension (10-15). To the best of our knowledge, a nomogram used in H-type hypertension, which is different from hypertension, has never been reported. Therefore, we aimed to explore the risk factors of H-type hypertension in the discrete population and demonstrate how a nomogram may be designed to predict the risk of H-type hypertension.

## MATERIALS AND METHODS

### Sample collection

This was a cross-sectional study, with samples obtained from Ningbo Medical Center LiHuili Hospital in Ningbo City, Zhejiang, China. A total of 1712 individuals (927 male, 785 female) who visited the hospital between January 2017 and December 2017 were recruited. The diagnostic criteria for H-type hypertension were as follows (16,17): (1) All patients with hypertension had at least three consecutive records of diastolic blood pressure (DBP) >90 mmHg and/or systolic blood pressure (SBP) >140 mmHg, or had received antihypertensive medications for more than 3 months; and (2) Hcy levels ≥10 μmol/L. The simple hypertension group comprised patients who only met the first diagnostic criterion. The HHcy group comprised individuals who only met the second diagnostic criterion, and the healthy controls were individuals with Hcy levels <10 μmol/L, SBP <120 mmHg, and DBP <80 mmHg and were not receiving antihypertensive therapy. In addition, all of the individuals were aged ≥18 years and had no history of secondary hypertension, diabetes mellitus, renal failure, myocardial infarction, stroke, drug abuse, or other serious diseases. The protocol of this study was approved by the Ethics Committee of Ningbo Medical Center LiHuili Hospital; all methods were carried out in accordance with approved guidelines, and written informed consent was obtained.

### Data collection and measurement

Sociodemographic data including age, sex, region, and occupation were collected. According to the standard specification, the height and weight of each participant were measured by professional medical examiners. Blood pressure was measured using a fully automatic electronic blood pressure monitor in the supine position twice ≥10 min apart by different trained technicians.

Fasting blood samples were extracted by venipuncture to measure the levels of blood lipids, including Hcy, glucose, uric acid, total cholesterol (TC), triglycerides (TGs), LDL, high-density lipoprotein (HDL), creatinine, aspartate aminotransferase (AST), and alanine transaminase (ALT). An ADVIA2400 automated biochemistry analyzer (Siemens AG, Munich, Germany) was used to measure blood lipids in a core laboratory with a standard protocol.

### Statistical analysis

Continuous variables, including age and BMI, and levels of TGs, glucose, TC, uric acid, HDL, LDL, Hcy, creatinine, AST, and ALT are described as means and standard deviations, and were compared using analyses of variances. Categorical variables such as sex were compared using Pearson’s chi-square test. Multiple logistic regression using the stepwise method (Forward wald) and a forest plot were applied to assess risk factors of H-type hypertension. A nomogram was constructed based on the results of the logistic regression to predict the occurrence of H-type hypertension. The nomogram comprised graphical lines corresponding to risk factors, points, total points, and risk of H-type hypertension. The length of the line of each risk factor reflected the regression coefficient estimated by logistic regression analysis.

The nomogram was evaluated from two aspects, namely, the discriminatory capacity and calibration ability. First, the discriminatory capacity was evaluated using Harrell’s C statistic concordance index (C-index) (18,19). The value of the C-index ranges from 0.5 (indicating a random chance) to 1.0 (indicating perfect concordance); generally, a C-index >0.7 is regarded as sound discrimination. Second, the calibration ability was evaluated using the calibration plot, which is a graph with a parametric definition (y: actual probability, x: predicted probability) (9). The actual probability is calculated by dividing the number of patients with the same predicted probability by the total patients, or calculated by grouping similar scores of predicted probabilities into subsegments when patients with the same probabilities are absent (20). The predicted probability, the prevalence rate, is calculated using the developed nomogram model. Additionally, the calibration plot has an ideal line drawn at a 45° angle, in which the actual probability and predicted probability are identical. If the graph’s equation is drawn along the ideal line, the developed nomogram is considered to have accurate predictive ability (9). Bootstraps with 1000 resamples were applied to both the C-index and calibration plot. All statistical analyses were carried out using PASW Statistics 19.0 (SPSS, Inc., Somers, NY, USA) and R software version 3.6.1 (http://www.R-project.org). A two-sided *p*<0.05 was considered statistically significant.

## RESULTS

### Characteristics of the study population

As shown in Table 1,712 subjects were included in this study: 282 patients with H-type hypertension (mean age, 47.02±11.77 years; 240 men), 105 patients with simple hypertension (mean age, 47.52±10.54 years; 49 men), 645 individuals with high Hcy levels (mean age, 39.99±10.01 years; 486 men), and 680 healthy controls (mean age, 38.05±8.23 years; 152 men). The prevalence rates of H-type hypertension in the total population, the hypertension population, and the HHcy population were 16.47%, 72.87%, and 30.42%, respectively. There were statistically significant differences in age, sex, BMI, and levels of TC, TGs, glucose, HDL, LDL, uric acid, creatinine, Hcy, AST, and ALT between the four groups (*p*<0.001).

### Logistic regression analysis of risk factors associated with H-type hypertension

#### H-type hypertension *vs*. healthy controls

**Figure 1:**
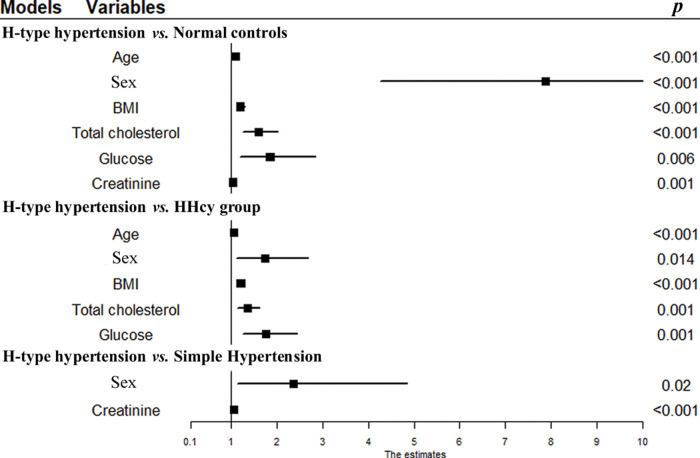
Forest plot based on logistic regression analysis. (*p* was adjusted for triglycerides, HDL, LDL, uric acid, AST, ALT, and/or age, BMI, total cholesterol, glucose, and creatinine).

Logistic regression analysis of age, sex, BMI, and levels of TC, TGs, glucose, HDL, LDL, uric acid, creatinine, AST, and ALT by forward selection showed that advanced age (odds ratio [OR], 1.087; 95% confidence interval [CI], 1.065-1.111; *p*<0.001], male sex (OR, 7.879; 95% CI, 4.270-14.537; *p*<0.001), high BMI (OR, 1.193; 95% CI, 1.107-1.286; *p*<0.001), high TC levels (OR, 1.591; 95% CI, 1.252-2.020; *p*<0.001), high glucose levels (OR, 1.843; 95% CI, 1.195-2.841; *p*=0.006), and high creatinine levels (OR=1.038; 95% CI, 1.015-1.061; *p*=0.001) were risk factors of H-type hypertension in the healthy population compared to healthy controls (Table 2, Figure 1).

#### H-type hypertension *vs.* HHcy group

Logistic regression analysis showed that advanced age (OR, 1.052, 95% CI: 1.036-1.069, *p*<0.001), male sex (OR, 1.730; 95% CI, 1.117-2.680; *p*=0.014), high BMI (OR, 1.202; 95% CI, 1.132-1.276; *p*<0.001), high TC levels (OR, 1.349; 95% CI, 1.134-1.605; *p*=0.001), and high glucose levels (OR, 1.749; 95% CI, 1.260-2.429; *p*=0.001) were risk factors of H-type hypertension in the HHcy population compared to the HHcy group (Table 2, Figure 1).

#### H-type hypertension *vs.* simple hypertension

Compared to those in the simple hypertension group, logistic regression analysis showed that male sex (OR, 2.354; 95% CI, 1.142-4.853; *p*=0.020), and high creatinine levels (OR, 1.055; 95% CI, 1.025-1.086; *p*<0.001) were risk factors of H-type hypertension in the hypertension population (Table 2, Figure 1).

### Nomogram construction and validation

**Figure 2:**
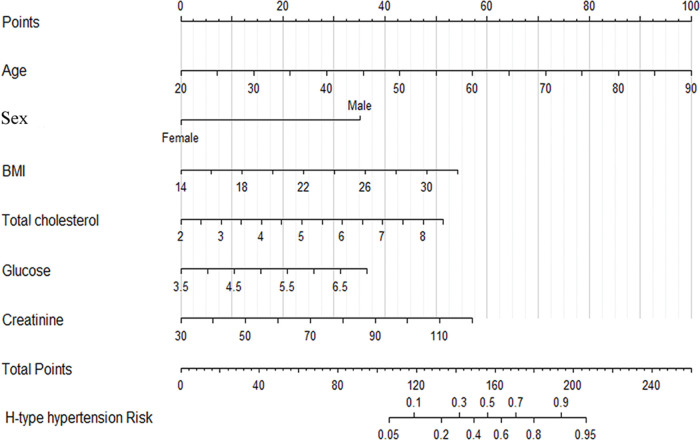
Nomogram to predict the probability of H-type hypertension. Each variable value is assigned a score, which is determined by drawing a vertical line to the points scale; the sum of these scores is located on the total points scale, and a vertical line is drawn downward to the predicted value scale to determine the probability of H-type hypertension.

As shown in Figure 2, a nomogram was constructed including six risk factors that were screened through logistic regression analysis. Each value of these factors was assigned a point, and the sum of these points was defined as the “total points,” which was converted to the probability of H-type hypertension in the lowest scale. In our nomogram models, the total points ranged from 106 to 206, and the corresponding risk rate ranged from 0.05 to 0.95 (Figure 2). Individuals with higher total points had a greater risk of H-type hypertension.

**Figure 3:**
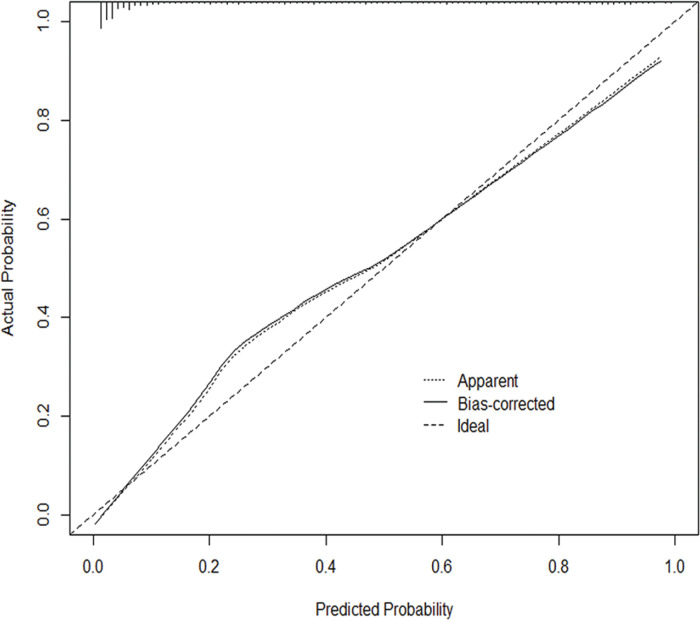
Calibration plot. The nomogram-predicted probability of H-type hypertension is drawn on the x-axis, and the y-axis represents the actual rate of H-type hypertension. The 45° line represents the ideal nomogram, where the predicted probability is the same as the actual probability. The solid line represents the bias-corrected line and the dotted line represents the apparent accuracy of the nomogram.

In addition, the C-index was 0.915 (95% CI: 0.897-0.933), indicating relatively good discriminative ability. The calibration plots (Figure 3) show that the predicted and actual probabilities were highly consistent. These results showed that the nomogram could accurately predict H-type hypertension in the studied population.

## DISCUSSION

Based on the comparisons of patients with H-type hypertension and healthy controls, H-type hypertension and HHcy, and H-type hypertension and simple hypertension, we found that advanced age, male sex, high BMI, high TC levels, high glucose levels, and high creatinine levels were risk factors of H-type hypertension in the healthy population; advanced age, male sex, high BMI, high TC levels, and high glucose levels were risk factors of H-type hypertension in the HHcy population; and male sex and high creatinine levels were risk factors of H-type hypertension in the hypertension population. Our findings indicated that men were more likely to have hypertension with HHcy, and that advanced age, high BMI, high TC levels, and high glucose levels mainly lead to hypertension; higher creatinine levels mainly lead to HHcy levels. Moreover, a nomogram model including age, sex, BMI, TC levels, glucose levels, and creatinine levels was constructed, which may be used as a predictor to identify and evaluate H-type hypertension risk early and intuitively and provide a basis for personalized prevention and treatment.

There are sex and age differences in H-type hypertension, and the risk of H-type hypertension is higher in men than in women and increases with age (21,22). Similarly, we found that the risk of H-type hypertension in men was 7.879 times (OR=7.879), 1.73 times (OR=1.73), and 2.354 times (OR=2.354) higher than that in women in the healthy, HHcy, and hypertension populations, respectively, indicating that men were more likely to have hypertension with HHcy. This difference may be due to the different physiological structures and lifestyles between the two sexes; men lack the effects of estrogen regulation on Hcy, which increases the prevalence of H-type hypertension in men (23,24). In addition, our results showed that the risk of H-type hypertension increased by 8.7% (OR=1.087) and 5.2% (OR=1.052) for every 1 year of age increase in the healthy and HHcy populations, respectively, but not in the hypertension population; this indicates that age may have a greater effect on blood pressure than Hcy levels. Therefore, greater attention should be paid to changes in blood pressure in elderly patients with H-type hypertension.

Moreover, various blood physiological indices have been found to be related to H-type hypertension. Wang et al. found that BMI and LDL levels are associated with H-type hypertension (1), while Han et al. indicated that uric acid, TG, and creatinine levels are associated with Hcy levels in patients with hypertension by logistic analysis (7). In the present study, high BMI, high TC levels, high glucose levels, and high creatinine levels were found to be risk factors of H-type hypertension in the healthy population; high BMI, high TC levels, and high glucose levels were risk factors of H-type hypertension in the HHcy population; and high creatinine levels were risk factors of H-type hypertension in the hypertension population. These results suggest that high creatinine levels mainly lead to high Hcy levels, possibly due to elevated creatinine levels causing renal dysfunction, which leads to a reduction in Hcy clearance and an increase in Hcy. Additionally, the difference in risk factors of H-hypertension in various studies may be due to the different pathophysiology and lifestyles among studied populations.

As a common complex and chronic disease, H-type hypertension is often affected by multiple factors. During its early intervention, knowing how to evaluate multiple factors simultaneously and intuitively is particularly important. In previous studies, researchers typically used methods such as logistic regression or Cox regression to evaluate the risk factors of H-type hypertension, but the interpretation of the results regarding multiple factors simultaneously was limited. Nomograms can simply show the probability of clinical events in statistical prediction models as scores through graphics, which can be used to evaluate multiple risks of diseases simultaneously and intuitively (8,9). Using this method, Chung et al. developed a nomogram for Koreans to predict the risk of type 2 diabetes using ten risk factors (14). Furthermore, a nomogram including age, BMI, parental hypertension, SBP, and DBP has also been constructed to predict the risk of hypertension in rural Chinese individuals (10).

Similarly, in the present study, we constructed a nomogram including age, sex, BMI, TC levels, glucose levels, and creatinine levels, which were screened through multivariate logistic regression analysis. Individuals with higher total points had a greater risk of H-type hypertension. For example, if an individual is 55 years of age, male, with a BMI of 24 kg/m^2^, TC level of 5.5 mmol/L, glucose level of 6 mmol/L, and creatinine level of 70 µmol/L, his total points is 193.5, and the corresponding risk of H-type hypertension is 90%; thus, the predicted probability of H-type hypertension of this patient can be regarded as very high (8,9). Through discriminatory capacity and calibration ability analysis, the results showed that this model was stable and had a high ability to predict H-type hypertension risk (C-index: 0.915, 95% CI: 0.897-0.933). To the best of our knowledge, this is the first study in which a nomogram for H-type hypertension has been designed. Consequently, our study provides intuitive and easy-to-understand clinical tools for precision medicine for H-type hypertension and provides a basis for personalized prevention and treatment.

In this study, the risk factors were corrected by logistic regression, and an intuitive and convenient graphical prediction model was constructed to obtain real and abundant results. However, there remain limitations. First, the present study was a cross-sectional study, and we could not determine the causal relationship between the risk factors and occurrence of H-type hypertension. Second, although the confounding factors were adjusted for by logistic regression, and the nomogram model was demonstrated to exhibit appropriate discriminatory capacity and calibration ability, the lack of smoking, alcohol consumption, intake of folic acid and vitamin B, and genotype may have affected the conclusions to some extent. Third, the participants were recruited from one hospital, and although the prevalence of H-type hypertension was similar to that in previous studies (25), whether the individuals are representative of other populations needs to be further validated. Therefore, a prospective multicenter cohort study must be performed, and data on smoking, alcohol consumption, and other important factors should be collected to compensate for the limitations in this study.

## CONCLUSIONS

Our findings suggest that H-type hypertension is associated with age, sex, BMI, TC levels, glucose levels, and creatinine levels. Advanced age, male sex, high BMI, high TC levels, and high glucose levels might lead to hypertension in the HHcy population, while male sex and high creatinine levels might lead to high Hcy levels in the hypertension population. The nomogram constructed in our study might be useful for both healthcare workers and laypeople for early, rapid, and intuitive evaluations of H-type hypertension risk and to provide a basis for personalized prevention and treatment.

## AUTHOR CONTRIBUTIONS

Fan R and Du J were responsible for the study design. Wang J and Du J were responsible for the data collection and interpretation. Fan R was responsible for the data analysis and manuscript preparation.

## Figures and Tables

**Table 1:** Characteristics of the study population (n=1712).

Characteristics	Normal controls (n=680) (mean±SD)	HHcy group (n=645) (mean±SD)	Simple hypertension (n=105) (mean±SD)	H-type hypertension (n=282) (mean±SD)	*F/χ^2^*	*p*
Age (years)	38.05±8.23	39.99±10.01	47.52±10.54	47.02±11.77	75.13	<0.001
Sex (M/F)	152/528	486/159	49/56	240/42	504.87	<0.001
BMI (kg/m^2^)	21.49±2.56	22.70±2.71	24.13±2.90	24.51±2.80	98.70	<0.001
Total cholesterol (mmol/L)	4.57±0.77	4.64±0.87	5.15±0.93	4.98±0.92	26.08	<0.001
Triglycerides (mmol/L)	1.07±0.60	1.40±1.05	1.57±0.98	1.73±1.19	39.39	<0.001
Glucose (mmol/L)	4.90±0.42	4.96±0.44	5.25±0.50	5.22±0.54	45.24	<0.001
HDL (mmol/L)	1.59±0.33	1.43±0.34	1.54±0.33	1.41±0.32	32.52	<0.001
LDL (mmol/L)	2.66±0.63	2.81±0.713	3.17±0.78	3.11±0.72	37.41	<0.001
Uric acid (mmol/L)	287.51±71.14	351.49±79.26	330.20±77.52	384.12±86.30	131.25	<0.001
Creatinine (µmol/L)	58.12±11.45	72.21±12.48	62.07±12.83	73.72±11.94	197.30	<0.001
Hcy (µmol/L)	8.00±1.46	14.54±8.72	8.21±1.56	15.48±11.22	131.18	<0.001
AST (U/L)	19.75±7.40	21.95±11.97	22.16±6.31	23.33±8.62	11.60	<0.001
ALT (U/L)	18.15±12.96	22.89±17.81	22.39±11.47	25.34±15.57	18.68	<0.001

**Table 2:** Logistic regression analysis of risk factors associated with H-type hypertension.

	H-type hypertension *vs.* normal controls		H-type hypertension *vs.* HHcy group		H-type hypertension *vs.* simple hypertension	
Variables	OR (95%CI)	*p*	OR (95%CI)	*p*	OR (95%CI)	*p*
Age	1.087 (1.065-1.111)	<0.001	1.052 (1.036-1.069)	<0.001	-	-
Sex	7.879 (4.270-14.537)	<0.001	1.730 (1.117-2.680)	0.014	2.354 (1.142-4.853)	0.020
BMI	1.193 (1.107-1.286)	<0.001	1.202 (1.132-1.276)	<0.001	-	-
Total cholesterol	1.591 (1.252-2.020)	<0.001	1.349 (1.134-1.605)	0.001	-	-
Glucose	1.843 (1.195-2.841)	0.006	1.749 (1.260-2.429)	0.001	-	-
Creatinine	1.038 (1.015-1.061)	0.001	-	-	1.055 (1.025-1.086)	<0.001

*p* was adjusted for triglycerides, HDL, LDL, uric acid, Hcy, AST, ALT, and/or age, BMI, total cholesterol, glucose, and creatinine.
